# Immunological Approaches to the Treatment of New Coronavirus Infection (Review)

**DOI:** 10.17691/stm2021.13.3.09

**Published:** 2021-06-28

**Authors:** N.A. Lyubavina, S.G. Saltsev, N.V. Menkov, L.V. Tyurikova, S.S. Plastinina, M.L. Shonia, A.A. Tulichev, M.Yu. Milyutina, E.V. Makarova

**Affiliations:** Associate Professor, Department of Propedeutics of Internal Diseases; Privolzhsky Research Medical University, 10/1 Minin and Pozharsky Square, Nizhny Novgorod, 603005, Russia; Associate Professor, Department of Propedeutics of Internal Diseases; Privolzhsky Research Medical University, 10/1 Minin and Pozharsky Square, Nizhny Novgorod, 603005, Russia; Associate Professor, Department of Propedeutics of Internal Diseases; Privolzhsky Research Medical University, 10/1 Minin and Pozharsky Square, Nizhny Novgorod, 603005, Russia; Associate Professor, Department of Propedeutics of Internal Diseases; Privolzhsky Research Medical University, 10/1 Minin and Pozharsky Square, Nizhny Novgorod, 603005, Russia; Associate Professor, Department of Propedeutics of Internal Diseases; Privolzhsky Research Medical University, 10/1 Minin and Pozharsky Square, Nizhny Novgorod, 603005, Russia; Associate Professor, Department of Propedeutics of Internal Diseases; Privolzhsky Research Medical University, 10/1 Minin and Pozharsky Square, Nizhny Novgorod, 603005, Russia; Assistant, Department of Propedeutics of Internal Diseases; Privolzhsky Research Medical University, 10/1 Minin and Pozharsky Square, Nizhny Novgorod, 603005, Russia; Assistant, Department of Propedeutics of Internal Diseases; Privolzhsky Research Medical University, 10/1 Minin and Pozharsky Square, Nizhny Novgorod, 603005, Russia; Associate Professor, Head of the Department of Propedeutics of Internal Diseases; Privolzhsky Research Medical University, 10/1 Minin and Pozharsky Square, Nizhny Novgorod, 603005, Russia

**Keywords:** COVID-19, SARS-CoV-2, coronavirus infection, pneumonia, lung damage, cytokine storm, immune response, prophylactic vaccines

## Abstract

The pandemic of the new coronavirus infection (COVID-19) caused by the SARS-CoV-2 virus has spread all over the world. The large amount of information that appears every day requires comprehension and systematization. The immunological aspects of the virus-host interaction are the core issues in the effective treatment and prevention of COVID-19’ development.

The review analyzes the known pathways of the viral invasion and evasion, the mechanisms of the cytokine storm, endothelial damage, and hypercoagulability associated with SARS-CoV-2 infection. Clinical data from previous SARS and MERS epidemics is discussed here. We also address the therapeutic approaches based on the basic knowledge of immune response and the blood cells’ immune functions, as well as the ways to reduce their hyperactivation. The use of interferon therapy, anti-inflammatory therapy, anti-cytokine therapy, neutralizing antibodies, convalescent plasma, and mesenchymal stem cells, as well as prophylactic vaccines, is discussed.

## Introduction

The pandemic of the new coronavirus infection (COVID-19) caused by the beta coronavirus SARS-CoV-2 has caused high morbidity and mortality worldwide [1]. The severity of COVID-19 ranges from asymptomatic to fatal pneumonitis [2]. SARS-CoV-2 is closely related to SARS (retrospectively named SARS-CoV-1) and the Middle East respiratory syndrome MERS-CoV, which caused zoonotic epidemics and localized outbreaks in 2003 and 2012, respectively [3–5]. Consequently, it is important to learn the lessons from the two previous coronavirus epidemics. Although SARS-CoV-2 is not nearly as deadly as SARS-CoV-1 or MERS-CoV [6], the rapid spread of the current infection has led to disastrous consequences for health systems around the world.

Numerous studies have shown that the penetration of the SARS-CoV-2 coronavirus into the cell is a result of interaction between the receptor-binding domain of the viral spike (S) protein and the angiotensin-converting enzyme receptor 2 (ACE2). The ACE2 is present on non-immune cells (respiratory and intestinal epithelial cells, endothelial cells, renal tubule cells, and brain neurons), as well as on immune cells (alveolar monocytes/macrophages) [7–9]. Binding of the viral S protein to the ACE2 receptor leads to the suppression of the latter followed by lung damage [10]. A better understanding of these immunological processes will allow to develop therapeutic and preventive measures against COVID-19 [11].

## Mechanisms of the immune response to SARS-CoV-2

### Innate immune response

The innate immune response serves as the first line of antiviral defense. There is a so-called mechanism of evasion, which allows them to evade the immune response, in coronaviruses. When the pattern-recognition receptors of immune cells interact with the viral RNA, the secretion of cytokines is triggered through signaling cascades. Among the cytokines, the most important for antiviral protection are type I and III interferons (IFN-I and IFN-III); in addition, other cytokines — pro-inflammatory tumor necrosis factor alpha (TNF-α), interleukins IL-1, IL-6, and IL-18 — are also released. Together, they induce an antiviral mechanism in target cells and potentiate the adaptive immune response. If it is initiated at an early stage and is powerful enough, IFN-I will be able to effectively limit the development of coronavirus infection and lead to pathogen elimination and patient recovery [12, 13]. Studies [14–17] have shown that SARS-CoV-2 is more sensitive to IFN-I and IFN-III than SARS-CoV-1 in laboratory conditions.

Cytokines represent a major barrier to viral infection; to avoid this barrier, coronaviruses have evolved mechanisms able to inhibit the induction and signaling of IFN-I. The SARS-CoV-2 virus suppresses the induction of intracellular interferon, while the virus is sensitive to exogenous IFN-I. Numerous studies [14, 18–22] have shown the ability of coronaviruses to inhibit interferon production *in vitro* and *in vivo*. In real clinical practice, patients with severe COVID-19 show a significant decrease in the effect of IFN-I compared to patients with mild to moderate cases of COVID-19 [23].

The existence of the pathways, especially that of IFN-I, that allow pathogenic coronaviruses to evade the immune response suggests a role for dysregulation of the IFN-I response in the pathogenesis of COVID-19. Pathogenic coronaviruses not only block interferon signaling but also participate in other inflammatory pathways. For example, the non-structural proteins NSP9 and NSP10 of SARS-CoV-2 can induce the production of IL-6 and IL-8 [24].

***Innate lymphoid cells*** are cytotoxic natural killer (NK) cells located in the lungs and able to prevent pathogens from entering the tissue. These cells do not express the ACE2 receptor and are, therefore, unlikely to be directly infected with SARS-CoV-2 [25]. Information of the number of NK cells in patients with COVID-19 is not consistent; yet these cells are assumed to play a role in the cytokine release syndrome [26, 27]. Studies [28, 29] indicate that in severe SARS-CoV-2 infections, the number of NK cells is reduced. After successful recovery from COVID-19, the NK cell number is restored to normal levels. Some reports [27, 30] indicate either a disturbance in the NK cell maturation or migration of mature circulating NK cells into the lungs or other peripheral tissues in patients infected with SARS-CoV-2.

An *ex vivo* study of NK cells from the peripheral blood of patients with COVID-19 revealed a decrease in the expression of CD107a, caspase Ksp37, granzyme B, and granulysin; these changes could result in reduced cytotoxicity and insufficient production of IFN-γ and TNF-α [31,32].

Patients with COVID-19 have increased plasma concentrations of IL-6 [32], which significantly correlated with a reduced number of NK cells [27, 33]. It has been shown that *in vitro* stimulation of IL-6 and its soluble receptor (IL6R) inhibits the cytolytic functions (production of perforin and granzyme B) of NK cells; this inhibition can be reversed by tocilizumab that blocks IL6R [34]. In addition, TNF-α secreted by monocytes regulates the differentiation of NK cells [35] and is able to bind to the specific receptors on NK cells [36]. The level of TNF-α was found to be increased in the blood plasma of patients with COVID-19 [32]. Thus, dysfunction of monocytes can negatively affect the antiviral function of NK cells in people infected with SARS-CoV-2.

***Monocytes/macrophages and dendritic cells*** play a crucial role in antiviral responses, linking innate and adaptive immunity. Peripheral activation and accumulation of activated pro-inflammatory monocytes/ macrophages in the lungs are recognized as one of the COVID-19 symptoms [37]. The SARS-CoV-2 virus activates monocytes/macrophages and triggers the production of pro-inflammatory mediators — IL-6, GM-CSF, IL-1β, TNF, CXCL-8, CCL-3 — and accelerated cell death. This can subsequently initiate a cytokine storm, which is also known as cytokine release syndrome. Some of these cytokines (for example, IL-6) are mainly secreted by macrophages, leading to macrophage activation syndrome [38–40]. Activated and subsequently dying macrophages contribute to high levels of ferritin in the blood plasma and dysregulation of iron metabolism. Abnormally high ferritin is one of the biomarkers for patients with severe COVID-19 [41, 42].

Thus, a therapy aimed at reducing the activity of macrophages may become promising means of suppressing the inflammatory storm during coronavirus infection.

Flow cytometric analysis of peripheral blood mononuclear cells from patients with advanced COVID-19 showed a significant rise in activated CD4+ T cells and CD14+HLA-DR^lo^ inflammatory monocytes known to produce granulocyte-macrophage colony-stimulating factor (GM-CSF) [43, 44]. Significantly increased systemic levels of the proinflammatory cytokine IL-6 were reported in several cohorts of COVID-19 patients; the levels of IL-6 correlated with disease severity [45]. Elevated levels of IL-6 might also be associated with high levels of IL-2, IL-7, IFN-γ, and GM-CSF found in secondary viral hemophagocytic lymphohistiocytosis (unregulated macrophage activation and unregulated phagocytosis). It is known that in response to viral infections, mononuclear cells increase their production of interleukins, IFN-I and IFN-III. This leads to the activation of inflammation, the induction of pathogenic reactions of Th1 and Th17 cells, the recruitment of effector immune cells, and the cytokine storm [46, 47].

Along with that, studies on SARS-CoV-2 and other coronaviruses showed that IL-6, IL-1β, and IFN-I/IFN-III from infected pulmonary epithelium are capable of inducing hyperinflammation cascades in resident (alternative) macrophages while simultaneously recruiting inflammatory monocytes, granulocytes, and lymphocytes from the bloodstream. This systemic inflammatory response can cause neutrophilic NETosis and microthrombosis, exacerbating the severity of COVID-19 [48].

***T-lymphocytes*** play a fundamental role in the antiviral response mediated by CD4+ T cell-secreted cytokines, CD8+ T cell cytotoxicity, and B cell activation, ultimately leading to the production of antibodies. New coronaviruses can bypass these protective mechanisms by inducing apoptosis of T cells, as well as depleting the pool of lymphocytes that enter the lungs and trigger hyperinflammation during the cytokine storm [49–52]. Dysregulation of T cell responses can lead to immunopathology.

It is important to clarify the role of T cells in the early immune response to the virus to understand the role of T cell responses. Previous studies on SARS-CoV-1 [53], as well as a number of recent studies on SARS-CoV-2 [27, 29, 54–56], indicate the development of lymphopenia with a sharply reduced number of both CD4 and CD8 T cells in moderate and severe clinical forms. The degree of lymphopenia, mainly CD8 T cells, in ICU patients correlated with the severity and mortality associated with COVID-19 [27, 39, 54, 57]. However, patients with mild COVID-19 usually have a normal or slightly increased number of T cells [58, 59]. The reason for the loss of peripheral T cells in moderate to severe COVID-19 needs further research since direct viral damage to T cells by SARS-CoV-2 was not found, in contrast to MERS-CoV [60].

Various mechanisms are likely to contribute T cells decrease in the blood, including the effects of inflammatory cytokines. Lymphopenia correlates with serum levels of IL-6, IL-10, and TNF-α, while recovery in the number of T cells along with lower levels of proinflammatory cytokines is noted in convalescent patients [29, 57, 61]. The cytokines IFN-I and TNF-α promote the retention of T cells in the lymphoid organs and their attachment to the endothelium [62, 63]. Autopsy findings indicated massive lymphocyte death in the spleen and enlarged intrathoracic lymph nodes of patients who died from COVID-19; a possible role of IL-6 and the Fas–FasL interactions was proposed. In support of this hypothesis, the IL-6 receptor antagonist tocilizumab was found to increase the number of circulating lymphocytes [43]. Recruiting T cells to the sites of infection can also reduce their presence in the peripheral blood. An increased number of CD8 T cells was found in the bronchoalveolar lavage fluid of patients with COVID-19 [37]. Similarly, autopsy of a patient with SARS-CoV-2 revealed extensive lymphocyte infiltration in the lungs [64], although another study found only neutrophil infiltration [65]. Therefore, further studies are needed to determine the cause and consequence of lymphopenia in patients with COVID-19.

In the study [39], it was suggested that dysregulation of T cell responses could provoke a more severe course of COVID-19. A significant increase in GM-CSF+ CD4 T cells with extremely high *ex vivo* IL-6 and IFN-γ production was reported in critically ill COVID-19 patients. In addition, a decrease in the level of regulatory T cells was noted in severe cases of the disease [28, 54].

Regulatory T cells help to resolve the inflammation in acute respiratory distress syndrome (ARDS) in mice [66]. A decrease in γδ T cells, a subset of T cells with protective antiviral function, was found in critically ill patients with COVID-19 [67]. Therefore, T cell deficit can lead to lung immunopathology in COVID-19.

Currently, little is known about specific functional changes in T cells associated with COVID-19. Most publications [36, 37, 58, 68, 69] report an increased presence of activated T cells characterized by the expression of HLA-DR, CD38, CD69, and CD25. As a rule, regardless of COVID-19 severity, the activation of CD8 T cells is more pronounced than that of CD4 T cells [28, 58]. Zheng et al. [29] noted that in critical forms of COVID-19, the number of polyfunctional T cells (producing more than one cytokine) was reduced in parallel with the production of IFN-γ, TNF-α, and granzyme B. Another study [69] demonstrated an increase in the granzyme B and perforin levels in CD8 T cells of critically ill patients.

In severe COVID-19 T cells seem to be overactivated and may become exhausted due to continuous expression of inhibitory markers, as well as decreased polyfunctionality and cytotoxicity. Conversely, there is an increase in follicular helper CD4 T cells in convalescent patients, as well as a decrease in the level of inhibitory markers, along with an increase in the level of effector molecules such as granzyme A, granzyme B, and perforin [29, 58].

In addition, memory T cells contribute to protective immunity during reinfection. Only a few studies addressed the specific T cell immunity in SARS-CoV-2 infection until now. A correlation was found between the neutralizing antibody titers and the number of virus-specific T cells [68] in mild COVID-19 convalescents. In another study, virus-specific CD4 and CD8 memory T cells were found in the peripheral blood of patients with moderate to severe ARDS [70].

Together, these proinflammatory processes are likely to lead to the cytokine storm developing in COVID-19 patients, which rationalizes the use of targeted immunosuppressive therapies. A clear understanding of the delicate balance between antiviral and inflammatory innate immune programs is essential for finding effective biomarkers and treatments for COVID-19.

### Humoral immune response

The humoral immune response is critical for the clearance of cytopathic viruses and plays a major role in the memory mechanism that prevents reinfection. SARS-CoV-2 induces a sustained B cell response, as evidenced by the rapid and almost universal detection of virus-specific IgM, IgG, and IgA, as well as neutralizing IgG antibodies, within days of infection. Kinetics of antibody response to SARS-CoV-2 is well described [71, 72].

Currently, studies on COVID-19 mainly focus on the IgG response to SARS-CoV-2 infection, because this line of research is highly important for the development of vaccines and therapeutic agents. IgG antibodies to various SARS-CoV-2 proteins can be determined in laboratory setting.

S and N protein-specific antibodies were assayed in serological studies [68, 73]. Antibodies against the S protein prevent the virus from binding to the host cells and are, therefore, able to effectively neutralize the virus. The presence of circulating IgG specific for the viral N protein is considered as a marker of previous infection. It was found out [74, 75] that the levels of anti-SARS-CoV-2-IgG against the viral nucleoprotein or the receptor-binding domain of the S protein correlated with the virus-neutralizing activity.

In previous epidemic outbreaks of coronavirus infections, serological responses to beta-coronaviruses varied. In SARS-CoV, specific IgG antibodies were found in all patients [76]; in milder forms of MERS-CoV, some patients did not show IgG at a sufficient level [77]. Currently, a number of studies of humoral immune responses [68, 71, 74, 78–80] report the appearance of IgG antibodies against SARS-CoV-2 in the blood serum of most patients. Some patients, mostly with mild forms of the disease, remained seronegative [81–83], which was likely associated with a short follow-up period (less than 25–50 days). A 90-day follow-up showed that IgG was not produced in 9% (3 of 32) of patients with mild symptoms of COVID-19, although all patients, including seronegative, showed neutralizing antibodies, indicating developed humoral immunity [84].

Therefore, seroconversion is likely to occur detected in all patients, but the sensitivity and specificity of IgG detection might be different.

Among those who had recovered after the SARS-CoV epidemic of 2003, the level of neutralizing antibodies remained significant for 3 years; in some reports, these antibodies persist to the present. Moreover, these persistent antibodies may have a neutralizing effect against the current coronavirus infection [85]. The duration of protective immunity against SARS-CoV-2 is currently unknown.

Information about the dynamics of the antibody response and serological status is ambiguous. Thus, Long et al. [78, 86] report that in 97% of 37 patients with mild COVID-19, the antibody titers decrease in 2–3 months after infection. According to Wajnberg et al. [87], the level of specific IgG remains stable for 82 days after the onset of symptoms. Marklund et al. [84] noted that the level of antibodies increased over time in some patients with either mild or severe forms of the disease. Xiao et al. [88] observed elevated levels of IgG in 34 patients with COVID-19 within 5 weeks of the onset of the disease; the values remained stable for 7 weeks. A number of reports [84, 89, 90] indicate that seroconversion occurs earlier among severely ill patients and specific antibodies are produced at a higher level. In addition, study [91] noted that gender did not significantly affect the serum level of anti-SARS-CoV-2 IgG in severe COVID-19.

The small number of published studies and the inconsistency of accumulated data do not allow us to draw final conclusions about the features and dynamics of humoral immunity in different forms of COVID-19. Further studies are required for the development of vaccines and pharmacotherapy, personalization and differentiation of approaches to immunization against this infection.

## Immunology-based approaches to therapy and specific prevention of COVID-19

### Interferon therapy

SARS-CoV-2 effectively inhibits the expression of IFN-I. Given its strong immunomodulatory nature, the administration of IFN-I to COVID-19 patients in the early stages of the disease may prevent the development of immunopathologies in the later stages. Numerous clinical trials on the use of interferon therapy have been launched. A study on efficacy of IFN-α-1β nasal drops combined with the immunomodulator thymosin α1 for the prevention of COVID-19 in high-risk medical staff is ongoing in China (NCT04320238, n=2944). A randomized clinical trial conducted in Iran [92] showed that the administration of IFN-β-1α to patients with severe COVID-19 reduced their hospital stay and 28-day mortality, especially with early treatment. The use of IFN-α2-β in combination with Arbidol significantly reduced the virus persistence in the upper respiratory tract and shortened the presence of the inflammatory markers IL-6 and CRP in adults hospitalized with COVID-19 [93] at the same time. However, these therapies increase the risk of over-activation of proinflammatory signals, which can enhance immunopathological manifestations [94].

Type III interferons can become an alternative to IFN-I because they also possess antiviral functions but are less toxic in terms of mediating immunopathology [95]. IFN-III–IFN-λ induces the production of IFN-γ by NK cells indirectly through IL-12, which partially suppresses and slows the immune response [96, 97]. A placebo-controlled study of pegylated IFN-λ is currently underway in patients with mild COVID-19 (NCT04331899, n=120). The main advantage of IFN-λ is that it can prevent the damaging effect of neutrophils on the lungs. On the other hand, it reduces the rate of tissue repair; in the context of COVID-19, which has a long time-course, this effect of IFN-λ may increase a risk of secondary infections [98]. Administration of interferons can cause an imbalance in the immune response and severe immunopathology in COVID-19. Therefore, careful monitoring of safety and efficacy of interferon therapy should be a priority in the development of protocols and clinical trials [99].

### The use of neutralizing antibodies and convalescent plasma for COVID-19 therapy

#### Neutralizing antibodies

The effectiveness of using neutralizing antibodies against SARS-CoV-1 and MERS-CoV has been documented [100, 101,102]. In the case of SARS-CoV-2, research efforts are primarily focused on identifying antibodies produced in diseased patients or under conditions of animal vaccination.

To obtain antibodies that neutralize the SARS-CoV-2 virus from the blood of recovered patients, Chinese scientists isolated memory B cells specific to the viral receptor-binding domain (RBD). Then these cells were cloned to express recombinant forms of the respective antibodies. The resulting four antibodies showed a high neutralizing potential *in vitro*. All of them inhibited the binding between ACE2 and RBD; however, blocking this interaction is not always a necessary condition for the effect of antibodies against SARS-CoV-2 [103].

Research is underway to obtain animal-raised antibodies that can be directly administered to SARS-CoV-2 infected patients. Regeneron Pharmaceuticals synthesized a combination cocktail of two antibodies REGN-COV2. The preparation is good at reducing the viral load when the immune response has not yet been manifested or the initial viral load is high [104]. AstraZeneca and Oxford University have developed a preparation of 2 monoclonal antibodies to the S protein of SARS-CoV-2 for the prevention of COVID-19 in adults. Therapy with anti-SARS-CoV-2 monoclonal antibodies seems promising since the medication can be used to block ongoing infection and as a prophylactic agent.

#### Convalescent plasma

Historically, passive immunotherapy used convalescent whole blood or convalescent plasma, human immunoglobulin, polyclonal or monoclonal antibodies. However, at present, the preference is given to plasma collected by apheresis [105]. The safety and efficacy of convalescent plasma in severe respiratory viral infections and severe COVID-19 are not fully studied [106, 107].

Studies conducted in a limited number of patients showed the potential efficacy of convalescent plasma transfusion as an adjunct treatment for severe COVID-19. The administration of 2 doses to 5 patients [108], 1 dose to 10 patients [109], and from 1 to 8 doses to 4 patients, including a pregnant woman [110], improved their clinical condition. A meta-analysis of 1 randomized controlled trial, 3 controlled and 10 uncontrolled clinical trials (5201 participants in total) on safety of convalescent plasma, revealed serious adverse events in the first 4 h after plasma transfusion. Other side effects were predominantly allergic or respiratory in nature, including anaphylaxis, shortness of breath, and acute lung injury. None of the studies reported adverse events in the control group [107].

The timing of convalescent plasma therapy is of great importance. In patients with SARS-CoV-1, the best results were observed with the administration of plasma before the 14^th^ day of illness [111], like it was found with influenza [112]. As shown, such therapy was more effective in PCR-positive, but seronegative patients, however, the amount of plasma and the frequency of transfusions require further study. The use of convalescent plasma from patients who have recovered from COVID-19 appears to be promising for the prevention of COVID-19 or when administered within 14 days after the onset of symptoms. Protection can last from several weeks to several months [109, 113, 114].

The results of a retrospective analysis of the use of convalescent plasma in 3082 adult patients treated in US hospitals for COVID-19 were published [115] in January 2021. A decrease in mortality was noted following the use of plasma with high titers (but not low titers) of specific antibodies in patients without mechanical ventilation. Initiation of anti-COVID plasma therapy up to 3 days after confirming the diagnosis of COVID-19 was associated with a lower mortality rate, in comparison to the later initiation of treatment.

Several non-randomized trials [108–110, 116, 117] showed the safety of convalescent plasma therapy and its beneficial effect in patients with severe COVID-19. Convalescent plasma has also been proposed for prophylactic use in people at risk (with comorbid pathology) or in medical staff who have been in contact with patients with COVID-19. The FDA has approved the use of convalescent plasma for the treatment of critically ill patients [118].

Other advantages of convalescent plasma include its availability and the seemingly low incidence of serious side effects. However, this cannot justify using a treatment with unproven efficacy. It is necessary to conduct randomized clinical trials to determine the optimal timing and indications for this therapy [119].

### Anti-inflammation therapy. Glucocorticoids

The host’s immune response plays a key role in the pathogenesis of severe COVID-19. Over time, it became obvious that lung damage in COVID-19 develops against the background of both overstimulation and suppression of the immune response [120]. Typically, there is a clinical picture of massive vascular inflammation, disseminated intravascular coagulation, shock, and ARDS [32, 120, 121]. Patients with severe COVID-19 face a double challenge. On the one hand, their fight against a viral infection necessitates the virus elimination from the body. On the other hand, the patients suffer from hyperinflammatory reactions, pulmonary thrombosis, increased vascular permeability, and ARDS [122]; those disorders necessitate the use of glucocorticoids (GCs) [123].

The anti-inflammatory effect of GCs can help overcome both hyperstimulation of the immune system and inflammation, as well as ARDS [123–126]. These medications are easily available and the treatment is cost-effective. However, the use of GCs was associated with the development of side effects in previous studies [127–132] with SARS-CoV-1 and MERS-CoV. Among them there was a slowdown in viral clearance, an increase in opportunistic infections, hyperglycemia, and suppression of the hypothalamic-pituitary-adrenal axis; those complications may limit the use of GCs in coronavirus infections. Therefore, during the SARS-CoV-2 pandemic, a large number of observational and randomized controlled trials were launched to study the efficacy of GCs against COVID-19.

Thus, preliminary results of the RECOVERY randomized trial [133] on dexamethasone therapy in hospitalized adults with confirmed COVID-19 were published in June 2020. For the first time dexamethasone was found superior to the standard treatment in reducing 28-day mortality in patients requiring oxygen therapy or mechanical ventilation.

In a prospective meta-analysis of seven randomized clinical trials, the administration of GCs was shown to be associated with a lower 28-day mortality from any cause [134]. WHO recommended against using GC medications in the early days of the pandemic. Currently, WHO proposes using systemic GCs for severe and critical forms of COVID-19, as well as in refractory shock and, if necessary, in patients with ARDS on mechanical ventilation [125, 135, 136].

In December 2020, Van Paassen et al. [137] published a systematic review and meta-analysis of 44 studies evaluating the efficacy of GC therapy in COVID-19, involving 20,197 patients aged 34 to 75 years. It was found that systemic GCs significantly had reduced short-term mortality (OR 0.72, 95% CI 0.57–0.87) and the need for mechanical ventilation in patients with acute respiratory failure (OR 0.71, 95% CI 0.54–0.97). However, it is too early to draw conclusions about the efficacy and safety of GCs in COVID-19. It is necessary to conduct large-scale studies where the indications for using GCs are clearly defined together with the consistent timing, dose, and duration of treatment.

### Anti-cytokine therapy

Some patients with SARS-CoV-2 infection develop pulmonary complications that can progress into ARDS and even more serious extrapulmonary systemic hyperinflammation syndrome [138].

Numerous studies [57, 61, 64, 139–142] have shown that hyperinflammation and cytokine storms, as well as increased levels of pro-inflammatory cytokines IL-6, -8, -2, -10, TNF-α, and IFN-α, correlate with the severity of COVID-19 and poor outcomes.

#### Monoclonal antibodies to IL-6

Following reports that IL-6 is a critical factor of the cytokine storm associated with COVID-19, monoclonal antibodies against IL-6 have been proposed as a therapeutic agent [141]. Clinical studies are underway to evaluate the efficacy of therapy with monoclonal antibodies against IL6R (tocilizumab, sarilumab, siltuximab) in pneumonia caused by COVID-19. Currently, only preliminary results are available.

Thus, Perrone et al. [143] showed that tocilizumab reduced the 30-day mortality with no significant toxic effects in patients who did not initially require mechanical ventilation. Xu et al. [144] studied patients with COVID-19 who received a single dose of tocilizumab combined with lopinavir, methylprednisolone, and oxygen therapy. Tocilizumab restored lymphocyte counts in 10 out of 20 patients and resolved pneumonia in 19 out of 20 patients (as determined by chest CT). All patients showed improved symptoms and there were not any secondary pulmonary infections.

In a retrospective study [145], a link was found between therapy with tocilizumab and a decrease in the likelihood of admission to the intensive care unit, as well as the need for mechanical ventilation. However, no significant reduction in mortality was observed in 30 patients with severe pneumonia associated with COVID-19.

It is reported [146] that phase III of the ongoing large-scale trial of sarilumab will be continued only in the “critical” group of patients (with improved outcomes) and not in the “severe” group.

In Russia, as it was recommended, targeted therapy with IL-6 inhibitors (tocilizumab, sarilumab) or IL-1β (canakinumab) in combination with GCs should be initiated to suppress the cytokine storm and prevent the development of severe lung damage and multiple organ failure [147].

***Granulocyte-macrophage colony-stimulating factor (GM-CSF)*** is a hematopoietic growth factor and a key mediator of tissue inflammation. GM-CSF has several cellular sources, including monocytes, macrophages, T cells, B cells, neutrophils, and tissue-resident cells [148]. GM-CSF has multiple pro-inflammatory effects on myeloid cells, including macrophages, monocytes, and neutrophils, by transmitting signals through the GM-CSF-α/β receptor complex that is expressed on myeloid cells. GM-CSF stimulates survival and facilitates polarization of the pro-inflammatory phenotypes of monocytes/macrophages, which primarily produce pro-inflammatory cytokines, including TNF-α, IL-1β, and IL-6. GM-CSF also increases the survival rate of neutrophils, stimulates their oxidative burst, enhances phagocytosis, promotes the adhesion of neutrophils to endothelial cells, and the transfer of neutrophils to inflammation sites [148]. GM-CSF is not detected in the healthy people’s circulating blood, but it was detected in the plasma of some patients hospitalized with COVID-19 [32]. It has been shown that the therapeutic inhibition of GM-CSF signaling by mavrilimumab in patients with rheumatoid arthritis reduces the level of IL-6 in the blood serum and indirectly suppresses the activation of T cells [149]. Treating patients with severe lung damage caused by COVID-19 using mavrilimumab — the monoclonal antibody to the GM-CSF receptor — resulted in good tolerability and clinical benefit (reduced fever and improved oxygenation).

Blocking GM-CSF can prevent immunopathology caused by the virus. The efficacy and safety of mavrilimumab and otilimab for COVID-19-associated lung damage are currently under study.

***Janus kinases*** regulate signal transduction into immune cells. Inhibition of cytokine production and Janus kinase activity by low molecular weight synthetic drugs makes it possible to block the cytokine storm [147]. It was found that the Janus kinase inhibitor baricitinib had the ability to inhibit the production of IL-6 [150]. However, this agent can lead to an increase in the number of NK cells, as it is shown among patients with rheumatoid arthritis treated with baricitinib [151], and, thus, adversely affect the condition of patients with severe COVID-19. Clinical trials of baricitinib, tofacitinib, and ruxolitinib in moderate COVID-19 patients are ongoing.

According to recommendations adopted in Russia, the use of Janus kinase inhibitors — baricitinib and tofacitinib — is possible for moderate pneumonia in order to suppress hyperinflammation and prevent the development of serious lung and other organ damage caused by COVID-19 [147].

#### Anakinra

Nod-like receptors play an important role in the innate immunity: they protect the body from a wide range of pathogens, including RNA viruses [152]. It is known that SARS-CoV induces the nod-like receptor NLRP3, which in turn stimulates caspase-1, a molecule involved in the activation and massive release of IL-1β and IL-18 [153]. These two can be successfully inhibited by anakinra, a recombinant antagonist of human IL-1 [154]. A study is underway (NCT04339712, NCT04330638) to test different dosing regimens of anakinra in COVID-19: from 100 mg/day subcutaneously for 28 days to 400–600 mg/day intravenously for 5–7 days.

***Colchicine*** is used to treat gout and familial Mediterranean fever. In recent years this drug has been used in the treatment of cardiovascular diseases to reduce the risk of ischemic complications [155]. A possible mechanism of action of colchicine in COVID-19 is its effect on cell adhesion molecules and inflammatory chemokines. Colchicine can inhibit the activation of NLRP3 in the inflammasome and also directly inhibit the synthesis of TNF-α and IL-6 [156]. Colchicine binds to the intracellular protein tubulin, which prevents the virus penetration into the cell nucleus and its subsequent replication; as a result, the viral load decreases [157]. The GRECCO-19 randomized study showed that treatment of patients with COVID-19 with colchicine helped reduce the time needed for normalization of the clinical condition, although no significant decrease in CRP levels was found [156]. The COLCORONA study [158] reported that the use of colchicine in outpatients with COVID-19 reduced the risk of hospitalization by 25%, death by 44%, and the need for ventilation by 50%.

Studies, including those in Russia, continue to test the efficacy of conventional therapeutic doses of colchicine in the treatment of COVID-19 (NCT04322682, NCT04328480, NCT04326790, NCT04403243).

#### Immune checkpoint inhibitors

In recent years there is an increasing therapeutic use of immune checkpoint inhibitors (ICIs) — antibodies that block the cytotoxic T-lymphocyte-associated protein 4 (CTLA-4), the protein of programmed cell death 1 (PD-1), and the ligand of programmed cell death receptor (PD-L1) [159]. Data on COVID-19-associated morbidity and mortality in cancer patients receiving ICIs are conflicting. A number of authors [160–162] noted that no increased susceptibility to SARS-CoV-2 infection was observed in these patients.

A growing body of evidence suggests that ICIs may be instrumental in treating viral infections by preventing T cell depletion. However, some cancer patients receiving ICIs require immune-suppressive therapy for autoimmune side effects, which in turn may aggravate the course of SARS-CoV-2 infection. In this regard, further research is needed to assess the effectiveness of this therapy in patients with COVID-19.

### Mesenchymal stem cells

Multipotent mesenchymal stem cells (MSCs) are present in most human tissues, including the umbilical cord. It is assumed that MSCs can reduce acute lung injury and inhibit the cell-mediated inflammatory response caused by SARS-CoV-2. MSCs lack the ACE2 receptor (used by the coronavirus to enter cells); therefore, they are resistant to infection [163, 164].

A pilot clinical study from China on intravenous MSCs transplantation involved 10 patients with confirmed COVID-19. Seven patients (1 with critically severe course, 4 with severe, and 2 with moderate COVID-19) received MSCs; 3 patients in serious condition — placebo. All patients who received MSCs recovered. Among the patients in the control group, one individual died, one developed ARDS, and one remained in a stable serious condition [165]. In another pilot study [166] 29 patients with severe COVID-19 received standard therapy (oxygen, umifenovir/oseltamivir, antibiotics as indicated, and GC); 12 patients received the same standard therapy and infusion of human umbilical cord MSCs (hUC-MSC). All patients who received hUC-MSC recovered and did not require mechanical ventilation. In 4 patients on standard therapy only, the condition worsened, they required mechanical ventilation, three of them died. However, the results are not statistically significant because the sample size was small.

In summary, it should be emphasized that an excessive inflammatory response with signs of a cytokine storm aggravates the course and worsens the prognosis in COVID-19. Numerous studies on SARS-CoV-2 and subsequent hyperinflammation are currently underway [167]. Medications used in daily rheumatologic practice may represent potential therapeutic options for COVID-19 due not only to their anti-inflammatory effect but also to some of their inherent antiviral properties.

### Prophylactic vaccines

SARS-CoV-2 is a new virus; therefore, the duration of protective immunity after infection is yet to be determined, although there is a possibility of a rapid decline in natural immunity [168].

A problem of vaccination against SARS-CoV-2 is that the vaccine-induced immune response can cause an acute reaction to the vaccine itself or an increase in infection severity upon contact with the virus; this mechanism could develop through an increase in T cells and antibody-dependent amplification syndrome [169]. In addition, some viruses use antibodies to enter target cells [170–172]. An increase in morbidity in vaccinated individuals who contracted an infection has already been observed, for example, with measles, respiratory syncytial virus, and Dengue virus [173–175].

By now the following types of vaccines against SARS-CoV-2 have been developed and undergo preclinical and clinical studies: inactivated; live attenuated; protein subunit; vector; based on synthetic virus-like particles, based on nucleic acids — DNA and RNA [168, 176, 177] (see the Table).

**Table:** Best known vaccine preparations (based on [168] and [177])

Types of vaccines	Name and manufacturer	Targeted viral antigen	WHO application status
mRNA-based	BNT162b2 (Pfizer, Inc., USA and BioNTech, Germany)	Spike	Approved
	mRNA-1273 (Moderna, USA)	Stabilized spike	Approved
	Zorecimeran (INN) concentrate and solvent for dispersion for injection (CureVac, Germany)	Spike	Under consideration
Vector	AZD1222 (AstraZeneca and University of Oxford, UK)	Spike	Approved
	Ad26.COV2.S (Janssen Pharmaceutica of Johnson & Johnson, Belgium, USA)	Spike	Approved
	Sputnik V (The Gamaleya National Center, Russia)	Spike	Under consideration
	Ad5-nCoV (CanSinoBIO, China)	Spike	Under consideration
Protein-based	EpiVacCorona (State Scientific Center of Virology and Biotechnology “Vector” of Rospotrebnadzor, Russia)	Multiple epitopes	Under consideration
	Recombinant Novel Coronavirus Vaccine (CHO Cell) (Zhifei Longcom, China)	RBD dimer	Under consideration
	SCB-2019 (Clover Biopharmaceuticals, China)	Trimeric S protein with alum adjuvant	Under consideration
	Cuba Soberana 01, Soberana 02, Soberana Plus (BioCubaFarma, Cuba)	Protein subunits	Under consideration
Inactivated	SARS-CoV-2 Vaccine (Vero Cell), Inactivated (lnCoV) (Sinopharm/Beijing Bio-Institute of Biological Products Co-Ltd, China)	Whole virus	Approved
	SARS-CoV-2 Vaccine (Vero Cell), Inactivated (Sinovac, China)	Whole inactivated virus	Under consideration
	COVAXIN (Bharat Biotech, India)	Whole virus	Under consideration
	Inactivated SARS-CoV-2 Vaccine (Vero Cell) (Sinopharm/ Wuhan Institute of Biological Products Co-Ltd, China)	Whole virus	Under consideration
Virus-like particles/nanoparticles	NVX-CoV2373/Covovax (Novavax, USA)	Spike	Under consideration

Most of the vaccines target the S protein of SARS-CoV-2. However, previous experience shows that the nucleocapsid (N) of the coronavirus is also immunogenic — antibodies against the N protein of SARS-CoV-1 are formed in a higher titer and persist longer than antibodies against protein S in recovered patients [178]. It is unknown if N protein is a potential protective immunogen for SARS-CoV-2, although vaccine formulations using the whole virus (inactivated, live attenuated vaccine) would potentially include N protein.

The first vaccine approved by WHO was BNT162b2 (Pfizer, Inc., USA and BioNTech, Germany). It consists of lipid nanoparticles modified with nucleosides of mRNA encoding the glycoprotein S of the SARS-CoV-2 virus [179].

Preclinical tests have shown both cellular and humoral immune responses against SARS-CoV-2 in mice [180] and humans (clinical phase I/II) — the formation of neutralizing antibodies upon a single vaccine administration [181]. A randomized phase II/III trial of 43,548 people showed that a two-dose regimen of BNT162b2 provided 95% protection against COVID-19 in people aged 16 and over. The 2-month safety of the vaccine was similar to that of other antiviral vaccines [182].

The second and third vaccines were developed by AstraZeneca and the University of Oxford (UK); those are produced in South Korea and India. AZD1222 is a vector vaccine based on the ChAdOx1 chimpanzee adenovirus carrying the coronavirus S protein gene. Preliminary results from the phase I/II study showed that seroconversion of neutralizing antibodies was observed in 91% of cases after one dose and 100% after two doses of the vaccine. An IFN-γ response has also been reported [183]. Humoral responses to the SARS-CoV-2 spike protein peaked on day 28 after vaccination and cellular responses were detected in all participants on day 14. Neutralizing antibodies were produced in all participants after the second dose of the vaccine. In addition, strong cellular and humoral immunogenicity was observed. Local and systemic adverse reactions of mild to moderate severity (pain at the injection site, chills, malaise, and headache) peaked on the first day of vaccination and were controlled with paracetamol [183].

The fourth vaccine which was recommended by WHO for use in emergencies is a single-ingredient preparation from Janssen Pharmaceutica of Johnson & Johnson (Belgium, USA).

According to the interim results of phase III clinical trials, the efficacy of the vector vaccine Ad26.COV2.S at a single dose was 66.1%, as assessed by preventing moderate and severe/critical forms of the disease and 85% in preventing severe/critical forms of the disease among all study participants from different regions, 28 days after vaccination. Anti-viral protection appears already on the 14^th^ day of vaccination [184].

The next (fifth) vaccine approved by WHO was mRNA-1273 (Moderna, USA) based on mRNA. Preclinical studies showed that it effectively protected mice against the virus [185]. In phase I of clinical trials, the vaccine showed immunogenicity and 100% seroconversion after the second dose [186]; the antibody titer increased with an increase in the administered dose. According to the results of a phase III study, the mRNA-1273 vaccine is 94.1% effective in preventing COVID-19. Mild, moderate, and severe systemic side effects have been identified; as reported, their severity significantly increases with the second vaccination and/or with the use of high doses of the vaccine. Antigen-specific T cells were most often found in the 100-μg vaccine group [187].

The sixth agent approved by the WHO against SARS-CoV-2 is the Vero Cell vaccine (Sinopharm, China) based on inactivated coronavirus. Phase III trials showed that its efficacy in preventing symptomatic disease and hospitalization is 79% for all age groups, which is lower than that of Pfizer or Moderna [188].

Three vaccines have been currently registered in Russia: Sputnik V (National Research Centre for Epidemiology and Microbiology named after Honorary Academician N.F. Gamaleya), EpiVacCorona (State Scientific Center of Virology and Biotechnology “Vector” of Rospotrebnadzor), and CoviVac (Chumakov Federal Scientific Center for Research and Development of Immune-and-Biological Products of Russian Academy of Sciences).

Sputnik V (Gam-COVID-Vac/Sputnik V) is a vector vaccine based on two different adenoviral vectors, Ad26 and Ad5 [189].

The results of phase I and II clinical trials show that Sputnik V is highly immunogenic and elicits strong humoral and cellular immune responses in 100% of healthy adult volunteers, while the antibody titers in vaccinated participants are higher than those in COVID-19 convalescents. The most common side effects were pain at the injection site (58%), fever (50%), headache (42%), asthenia (28%), and muscle/joint pain (24%). Most adverse events were mild and less pronounced with the administration of the freeze-dried vaccine [189].

An interim analysis of phase III clinical trials showed that the vaccine efficacy was 91.6% (95% CI 85.6–95.2), including people over 60 years old. According to [190], Sputnik V protects 100% against moderate and severe COVID-19.

The EpiVacCorona vaccine consists of chemically synthesized peptides identical to the S protein immunogens of the SARS-CoV-2 coronavirus, conjugated to a carrier protein, and adsorbed on aluminum hydroxide. The results of phase I and II clinical trials showed that in 100% of volunteers, vaccination caused the development of antibodies specific to the antigens that make up the vaccine. Seroconversion (with a neutralizing antibody titer ≥1:20) was confirmed in 100% of cases 21 days after the second vaccination [191].

CoviVac is the latest Russian development based on the platform of inactivated children’s poliomyelitis vaccine. CoviVac not only blocks the viral S protein but mimics the body’s natural process of fighting the virus. The CoviVac preparation is based on the whole virus, therefore, it is effective against most variants or mutations of SARS-CoV-2 [192].

Recent publications have revealed evidence of the so-called trained immunity that could protect against COVID-19. This principle is based on the non-specific enhancement of immune responses using the BCG vaccine (Mycobacterium bovis Bacillus Calmette–Guérin) or other bacterial components. The ability of BCG vaccine to induce “trained” immunity and stimulate antiviral immune response has been shown in animal experiments and clinical trials. Several countries are conducting randomized clinical trials in vulnerable populations to study the possibility of increasing protection against COVID-19 through BCG vaccination. In addition, it is important to determine which BCG strain and which vaccine can produce the strongest immunity [193, 194].

It is too early to draw conclusions on the effectiveness of anti-SARS-CoV-2 vaccines and their role in reducing the burden of the pandemic.

## Conclusion

The rapid spread of SARS-CoV-2 and the outbreak of the pandemic around the world have become a reason for appearing of a large number of scientific papers shedding light on the immunology of the new coronavirus infection. In many of these publications, studies on the previous outbreaks of infections associated with SARS-CoV-1 and MERS-CoV have been taken into consideration. However, the immune responses to SARS-CoV-2 are different from those seen in other coronavirus infections. Thus, in contrast to the previous two pathogens, a large number of infected individuals remain asymptomatic, the incubation period lasts longer, and the transmission rate is higher than with other coronaviruses. Consequently, a clear understanding of the immunological aspects of the virus-host interaction is needed to develop means of effective treatment and prevention of COVID-19.
